# Clinical coding of prospectively identified paediatric adverse drug reactions – a retrospective review of patient records

**DOI:** 10.1186/2050-6511-15-72

**Published:** 2014-12-17

**Authors:** Jennifer R Bellis, Jamie J Kirkham, Anthony J Nunn, Munir Pirmohamed

**Affiliations:** Research & Development, Alder Hey Children’s NHS Foundation Trust, Liverpool, UK; Department of Biostatistics, University of Liverpool, Liverpool, UK; Department of Women’s & Child Health, University of Liverpool, Liverpool, UK; Department of Molecular and Clinical Pharmacology, University of Liverpool, Liverpool, UK

**Keywords:** Adverse drug reaction, Paediatrics, Medical coding

## Abstract

**Background:**

National Health Service (NHS) hospitals in the UK use a system of coding for patient episodes. The coding system used is the International Classification of Disease (ICD-10). There are ICD-10 codes which may be associated with adverse drug reactions (ADRs) and there is a possibility of using these codes for ADR surveillance. This study aimed to determine whether ADRs prospectively identified in children admitted to a paediatric hospital were coded appropriately using ICD-10.

**Methods:**

The electronic admission abstract for each patient with at least one ADR was reviewed. A record was made of whether the ADR(s) had been coded using ICD-10.

**Results:**

Of 241 ADRs, 76 (31.5%) were coded using at least one ICD-10 ADR code. Of the oncology ADRs, 70/115 (61%) were coded using an ICD-10 ADR code compared with 6/126 (4.8%) non-oncology ADRs (difference in proportions 56%, 95% CI 46.2% to 65.8%; p < 0.001).

**Conclusions:**

The majority of ADRs detected in a prospective study at a paediatric centre would not have been identified if the study had relied on ICD-10 codes as a single means of detection. Data derived from administrative healthcare databases are not reliable for identifying ADRs by themselves, but may complement other methods of detection.

**Electronic supplementary material:**

The online version of this article (doi:10.1186/2050-6511-15-72) contains supplementary material, which is available to authorized users.

## Background

National Health Service (NHS) hospitals in the UK use a system of coding alongside the length of hospital stay to determine the chargeable cost of care for each patient. The coding system used is the International Classification of Disease (ICD-10). The process of coding is by case note review, undertaken by trained coders. It relies on diagnoses, procedures and other events being written down by the clinical team caring for the patient. The data are submitted to become part of the national hospital episode statistics (HES) for the NHS in England and are used for research and planning.

There are ICD-10 codes which may be associated with adverse drug reactions (ADRs). Although these codes can also be associated with overdose and medication errors, there is a possibility of using ICD-10 codes for ADR surveillance. Previous studies in the adult population have identified ADRs or ADEs either by retrospective case note review or by prospective monitoring, and then reviewed the ICD-10 codes for these cases; these studies showed that, in the majority of cases, ADRs and ADEs had not been coded properly [1–3]. Other studies have used ICD-10 codes to determine ADR or ADE incidence followed by a review of the case note record to confirm the accuracy of the code. This method can identify false positives but does not allow the investigator to estimate how many ADRs have been missed by using ICD codes for their identification [4–7]. A previous paediatric study identified ADR cases by searching for the relevant ICD codes and compared these with spontaneously reported cases. They found that not all ADRs were identified by both methods and concluded that neither method in isolation was reliable for ADR surveillance [8].

In this study, we aimed to determine whether prospectively identified ADRs in a paediatric population could have been detected via review of ICD-10 codes.

## Methods

The electronic records of children in whom a total of 241 ADRs had been prospectively identified in an earlier study, were reviewed. The aim was to determine whether an ICD-10 code related to the ADR had subsequently been added to their electronic admission abstract.

The methodology of the prospective study in which the 241 ADRs were initially detected has been described previously [9], here a brief summary is provided. The study duration was 12 months (2008–2009) and the participants were children admitted to a paediatric tertiary referral centre (Alder Hey Children’s NHS Foundation Trust). A prospective review of each unplanned admission was undertaken by a member of a multi-disciplinary research team which comprised a paediatrician, a paediatric nurse and a pharmacist. The total number of reviews undertaken by each team member and the types of patients in each caseload were similar. The research team reviewed medication taken in the two weeks prior to admission alongside the reason for admission to determine whether the admission was partly or entirely related to an ADR. The severity, causality and avoidability of each suspected ADR were subsequently evaluated by the research team. The records of the patients identified in this study as having experienced an ADR were examined in order to undertake the retrospective study described below.

For each ADR, the following information was obtained from the prospective study dataset to meet the specific objectives of this retrospective study: patient identification, suspected drug(s), ADR description (usually a symptom), ADR type, severity and causality. ADR type was defined as either Type A (augmented) resulting from an exaggeration of a drug’s normal pharmacological actions or Type B (bizarre) which are novel responses that are not expected from the known pharmacological actions of the drug [10]. ADR severity was classified according to an adaptation of the Hartwig scale [11]; 1 - No change in treatment with suspected drug, 2 - Drug dosing or frequency changed, without antidote or treatment, 3 - Required treatment, or drug administration discontinued, 4 - Resulted in patient transfer to higher level of care, 5 - Caused permanent harm or significant haemodynamic instability, 6 - Directly or indirectly resulted in patient death. ADR causality was assessed using the Liverpool Causality Assessment Tool (LCAT) and the outcome could be unlikely, possible, probable or definite [12].

The electronic admission abstract for each patient identified through the earlier prospective study as having experienced at least one ADR was examined and a record was made of whether the ADR(s) and/or their signs and symptoms had been coded using ICD-10 and if so, which code(s) had been used.

At Alder Hey, clinical coding is undertaken by a non-clinical team of trained individuals. There were two types of code relevant to ADRs: external cause codes Y40-Y59 (adverse effects in therapeutic use) for example, *‘Y42 Hormones (including synthetic, antagonists)’* (Table 1) and codes including the word ‘drug induced’ for example: *‘T88.6 Drug-induced anaphylaxis’, ‘E16.0 Drug induced hypoglycaemia without coma’*. Codes for drug-induced complications were listed in the relevant chapters of the ICD-10 handbook according to the system affected – for example *‘E16.0 Drug induced hypoglycaemia without coma’* was listed in Chapter IV: Endocrine, nutritional and metabolic diseases [13]. We refer to all of these here as ‘ICD-10 ADR codes’. A record was made of whether the ADR signs and symptoms had been coded using ICD-10 with no acknowledgment of their drug cause, for example a drug-induced rash recorded as *‘D69.0 Allergic purpura’.* We refer to these as ‘ICD-10 sign and symptom codes’.

**Table 1:** **ICD-10 codes which may apply to adverse drug events or adverse drug reactions**

Code	Description
Y40	Systemic antibiotics
Y41	Other systemic anti-infectives/antiparasitics
Y42	Hormones (including synthetic, antagonists)
Y43	Primarily systemic agents
Y44	Agents primarily affecting blood constituents
Y45	Analgesics/antipyretics/anti-inflammatories
Y46	Antiepileptics/antiParkinsonism drugs
Y47	Sedatives, hypnotics, antianxiety drugs
Y48	Anaesthetics, therapeutic gases
Y49	Psychotropic drugs
Y50	CNS stimulants
Y51	Drugs affecting autonomic nervous system
Y52	Agents primarily affecting cardiovascular system
Y53	Agents primarily affecting gastrointestinal system
Y54	Agents affecting water/mineral balance/uric acid
Y55	Agents affecting muscle/respiratory system
Y56	Topical agents affecting skin, ENT, dental
Y57	Other and unspecified medicaments
Y58	Bacterial vaccines
Y59	Other vaccines/biologicals

Y40-Y59 external cause codes (adverse effects in in therapeutic use).

A Chi-square test for difference in proportions was used to determine whether there were any differences in the characteristics of coded and uncoded ADRs.

This study used routinely collected clinical data in an anonymized format. The Chair of the Liverpool Paediatric LREC informed us that this study did not require individual patient consent or review by an Ethics Committee.

## Results

Of the 241 ADRs evaluated in this retrospective study, 76 (31.5%) were coded using at least one ICD-10 ADR code, two reactions had two codes (Table 2). One reaction was incorrectly coded; a skin reaction to topical dimeticone, was coded as ‘*Y53.1 Other antacids and anti-gastric-secretion drugs’.*

**Table 2:** **ADRs coded using ICD-10 ordered by reaction frequency (n = 76, two reactions had two codes)**

***Description of reaction (s)***	***ICD -10 code***	***Number of reactions***
ADRs secondary to chemotherapy: neutropenia, anaemia, thrombocytopenia, immunosuppression, deranged liver function tests, mouth ulcers, nausea, vomiting, diarrhoea, back pain, fever, deranged renal function	Y43.3 Other antineoplastic drugs	70
Rash secondary to penicillin Vomiting secondary to penicillin	Y40.0 Penicillins	2
Hyperglycaemia secondary to dexamethasone	Y42.7 Androgens and anabolic congeners	2
Anaemia, immunosuppression, thrombocytopenia, neutropenia	Z51.2 Other chemotherapy	1
Hypoglycaemia secondary to insulin	E16.0 Drug induced hypoglycaemia without coma	1
Irritability following pneumococcal and DTP vaccines	T88.1 Other complications following immunization	1
Irritability following pneumococcal and DTP vaccines	Y59.9 Vaccine or biological substance, unspecified	1

A large proportion of ADRs in the prospective study [2] occurred in patients under the care of the oncologists; we refer to these as oncology ADRs and to the remaining ADRs as non-oncology. There were 126 non-oncology ADRs and 115 oncology ADRs. Of the 126 non-oncology ADRs, 6 (4.8%) were coded using an ICD-10 ADR code. Of the 115 oncology reactions, 70 (61%) were coded using an ICD-10 ADR code (difference in proportions 56%, 95% CI (46.2% to 65.8%); p < 0.001). Without exception, the code ‘*Y43.3 other antineoplastic drugs’* was used to indicate adverse effects which had occurred secondary to the therapeutic use of oncology drugs.

The signs and symptoms of 212/241 (88%) ADRs were acknowledged by ICD-10 sign and symptom code(s). These 212 ADRs cases were made up of 107 oncology cases of which 70 also had an ADR code and 104 non-oncology cases of which 4 also had an ADR code. There were a variety of ICD-10 codes used for the same reaction type (Additional file 1: Table S1). There were 20/126 (15.9%) non-oncology ADRs and 8/115 (7.0%) oncology ADRs not acknowledged by either an ICD-10 ADR code or an ICD-10 sign and symptom code.

Considering oncology and non-oncology reactions together, coded ADRs were not more likely to be type A than type B reactions (difference in proportions 3%, 95% CI: -3.0% to 8.9%, p = 0.26). Coded ADRs were not more likely to be of severity 1, 2 and 3 than those of severity 4 and 5 (difference in proportions 6%, 95% CI -0.1% to 11.9%, p = 0.07). Finally, coded ADRs were more likely to be definite and probable ADRs than possible ADRs (difference in proportions 47%, 95% CI 32.3% to 59.7%; p < 0.001).

## Discussion

This retrospective study of electronic patient records demonstrates that the majority of ADRs detected in a prospective cohort study at a paediatric tertiary care centre would not have been identified if the study had relied on ICD-10 codes as a single means of detection. The use of ICD10 ADR codes was infrequent. The use of ICD-10 sign and symptom codes was more frequent but a wide variety of codes were used for the same ADR type. When undertaking research which uses data derived from administrative healthcare databases it is essential to first consider the reliability of those data.

The limitations of this study relate to those of the prospective cohort study in which the ADR cases were identified. The recording of a medication history for each participant relied on parents and/or patients recalling and communicating accurately all medicines administered prior to admission. Clearly there was scope for errors and omissions in this process. The detection of suspected ADRs by the study team relied on two things: a) signs and symptoms associated with the ADR being recorded by the clinical team looking after the patient; and b) the study team suspecting a link between signs and symptoms recorded and the medicines administered before admission. Where signs and symptoms were not recorded or the study team missed the link, the ADR was not highlighted and an evaluation of how it had been coded could not be undertaken.

In previous studies of ICD-10 coding for ADRs and ADEs in the adult population, the reported rate of coding was 2.0-6.8% [1, 2]. Here, a higher rate of ICD-10 codes being used to record ADRs (31.5%) was found. This can be explained by the high proportion of oncology ADRs that were coded using an ICD-10 ADR code. The two main reasons for this were: a) the oncology unit was using a structured admission proforma for unplanned admissions presenting with febrile neutropenia (one of the most common ADRs in this study); this proforma was also used to record details of other drug-related problems and b) there were specifically trained, non-clinical, coding staff assigned to the oncology unit. If we consider the non-oncology ADRs only, the rate of ADR coding (4.8%) is more comparable to that in adult studies.

As a result of the system by which coding is undertaken, there are reasons why ADRs may not be coded using an ICD-10 ADR code. An ADR cannot be coded if it is not identified and subsequently recorded by the clinical team; this relies on many factors including the ability of the clinical team to identify an ADR and record it as such. It is interesting to note that ADRs which were classified as probable or definite in this study were more likely to have been coded using an appropriate ICD-10 ADR code. This suggests that a more confident causality assessment by the clinician is more likely to lead to a case-note entry which is picked up by coders.

Many ADRs which were not coded using an ICD-10 ADR code had their signs and symptoms coded (Additional file 1: Table S1). The ICD-10 sign and symptom codes for each reaction were recorded to explore whether any of these codes were commonly being used for ADR cases and if so, whether they could provide an additional means of ADR detection. However, there was inconsistency in coding. For example, amongst the non-oncology ADRs, 15 different ICD-10 codes were associated with 18 cases of immunosuppression. The diversity of sign and symptom codes used would have limited their usefulness for the detection of ADRs in our cohort of patients.

## Conclusions

In summary, the use of ICD-10 codes to identify ADRs leading to admission at a paediatric tertiary care centre is not currently a reliable method of pharmacovigilance but may complement other approaches. A standardised approach to the use of administrative healthcare data for the identification of adverse drug events has the potential to improve the reliability of this method [14].

## Electronic supplementary material

Additional file 1: Table S1: ADRs only acknowledged by ICD 10 signs and symptom codes. (DOCX 20 KB)
