# Supplementary material for: Clinical coding of prospectively identified paediatric adverse drug reactions – a retrospective review of patient records
Source: BMC Pharmacol Toxicol. 2014 Dec 17;15:72. doi: 10.1186/2050-6511-15-72 (PMC4290086; doi:10.1186/2050-6511-15-72)
Supplement: Supplementary file 1 — Additional file 1: Table S1: ADRs only acknowledged by ICD 10 signs and symptom codes. (DOCX 20 KB) [file 40360_2014_357_MOESM1_ESM.docx]

Supplementary Table 1 ADRs only acknowledged by ICD 10 signs and symptom codes, ordered by reaction frequency (n=137)

| **Reaction** | **ICD-10 code for signs and or symptoms** | **Number of reactions** |
| --- | --- | --- |
| Post-tonsillectomy bleed | T81.0 Haemorrhage and haematoma complicating a procedure NEC  K92.0 haematemesis  H95.8 other post procedural disorders of ear and mastoid process | 27 |
| Immunosuppression | Z94.8 Other transplanted organ and tissue status  Z94.4 Liver transplant status  B00.9 Herpes viral infection, unspecified  A41.9 Septicaemia, unspecified  Z94.2 Lung transplant status  Z94.0 Kidney Transplant status  R50.9 Fever, unspecified  N39.0 Urinary tract infection, site not specified  M72.58 Fasciitis nec, other head neck ribs skull trunk vertebral column  L30.9 Dermatitis unspecified  J02.9 Acute pharyngitis, unspecified  B96.8 Other specific bacterial agents as cause of disease  B02.9 Zoster without complication  B01.9 Varicella without complication  A40.9 streptococcal septicaemia, unspecified  R50.9 fever, unspecified  Z94.2 Lung transplant status  J22.X Unspecified acute lower respiratory infection  K61.0 Anal abscess  B08.1 Molluscum contagiosum  J22.X Unspecified acute lower respiratory infection  Z94.9 other transplanted organ and tissue status  J06.9 Acute upper respiratory infection, unspecified | 23 |
| Constipation | R32.X Unspecified urinary incontinence  R10.4 Other and unspecified abdominal pain  K62.5 Haemorrhage of anus and rectum  K59.0 Constipation  R10.4 Other and unspecified abdominal pain.  R11.X Nausea and vomiting  K59.0 Constipation | 7 |
| Rash | R23.3 Spontaneous ecchymoses  R21.X Rash and other nonspecific skin eruption  D69.0 Allergic purpura  L03.1 Cellulitis of other parts of limb | 6 |
| Hypoglycaemia | E16.2 Hypoglycaemia, unspecified | 6 |
| Thrombocytopenia, neutropenia | R50.9 Fever, unspecified  R04.0 Epistaxis | 3 |
| Seizure | R56.8 Other and unspecified convulsions  R56.0 Febrile convulsions  G41.9 Status epilepticus | 3 |
| Respiratory depression | R09.2 Respiratory arrest  R06.0 Dyspnoea  E85.2 Non-invasive ventilation | 3 |
| Neutropenia | X90.3 Neutropenia drugs band 1  R50.9 Fever, unspecified  D70.X Aranulocytosis | 3 |
| Vomiting | R11.X Nausea and vomiting | 2 |
| Thrombocytopenia | D69.3 Idiopathic thrombocytopenic purpura  D69.6 Thrombocytopenia, unspecified | 2 |
| Neutropenia, immunosuppression | A08.0 Rotaviral enteritis  D70.X Agranulocytosis  R50.9 fever, unspecified | 2 |
| Immunosuppression, deranged LFTs | J22.X Unspecified acute lower respiratory infection | 2 |
| Headache | G93.2 Benign intracranial hypertension  R51.X Headache | 2 |
| Haematemesis | K29.7 gastritis, unspecified  K29.0 Acute haemorrhagic gastritis | 2 |
| Fever, seizure | R56.0 Febrile convulsions | 2 |
| Fever | R50.9 Fever, unspecified | 2 |
| Diarrhoea | K52.9 Noninfective gastroenteritis and colitis, unspecified  A08.4 Viral intestinal infection, unspecified | 2 |
| Anaemia, thrombocytopenia, neutropenia | R50.9 Fever, unspecified  D70.X Agranulocytosis | 2 |
| Wheeze, increased work of breathing | B34.9 Wheezing | 1 |
| Vomiting, neutropenia, immunosuppression, diarrhoea, thrombocytopenia, deranged LFTs | D70.X Agranulocytosis | 1 |
| Vomiting, diarrhoea, difficulty in breathing | K52.9 Noninfective gastroenteritis and colitis, unspecified | 1 |
| Vomiting, abdominal pain | R21.X Rash and other nonspecific skin eruption  R11.X nausea and vomiting | 1 |
| Thrombocytopenia, immunosuppression, neutropenia | D70.X Agranulocytosis  R50.9 fever, unspecified | 1 |
| Thrombocytopenia, anaemia, deranged LFTs, vomiting, nausea, diarrhoea | K52.9 Noninfective gastroenteritis and colitis, unspecified | 1 |
| Seizure, respiratory depression | R56.0 Febrile convulsions | 1 |
| Renal dysfunction | N28.9 Disorder of kidney and ureter, unspecified | 1 |
| Rash, irritability, fever | B34.9 viral infection, unspecified | 1 |
| Rash, fever, lethargy | R50.9 Fever, unspecified  R23.3 Spontaneous ecchymoses | 1 |
| Pyrexia, vomiting | R50.9 Fever, unspecified, R11.X nausea and vomiting | 1 |
| Pyrexia, irritability | J06.9 Acute upper respiratory infection, unspecified | 1 |
| Post-operative bleeding | T81.0 Haemorrhage and haematoma complicating a procedure NEC | 1 |
| Neutropenia, thrombocytopenia, anaemia, immunosuppression | K13.7 Other and unspecified lesion of oral mucosa | 1 |
| Neutropenia, gastritis | K29.7 Gastritis, unspecified | 1 |
| Mucositis, neutropenia, anaemia, thrombocytopenia | D70.X agranulocytosis  R50.9 fever, unspecified  K12.1 Other forms of stomatitis | 1 |
| Limb swelling | L03.1 Cellulitis of other parts of limb | 1 |
| Leukencepalopathy | R11.X Nausea and vomiting  G81.9 Hemiplegia unspecified  R29.8 Other specific signs involving nervous/musculoskeletal systems | 1 |
| Kawasaki disease | M30.3 Mucocutaneous lymph node syndrome | 1 |
| Irritability | R68.1 nonspecific symptoms peculiar to infancy | 1 |
| Intestinal obstruction | J56.0 Paralytic ileus | 1 |
| Intermenstrual bleed | N92.0 Excessive and frequent menstruation with regular cycle | 1 |
| Impaired healing | T81.3 Disruption of operation wound, not elsewhere classified | 1 |
| Immunosuppression, low lymphocyte count | B02.9 Zoster without complication  B01.9 varicella without complication | 1 |
| Immunosuppression, anaemia | R30.0 Dysuria | 1 |
| Ileus | K56.7 Ileus unspecified | 1 |
| Hypertension | I10.X Essential (primary) hypertension | 1 |
| Hyperglycaemia | E13.8 Other specified diabetes mellitus with unspecified comps | 1 |
| Headache, thrombocytopenia, neutropenia, anaemia, diarrhoea, vomiting | D70.X Agranulocytosis  R50.9 fever, unspecified | 1 |
| Haematuria, thrombocytopenia, anaemia | R31.X Unspecified haematuria | 1 |
| Diarrhoea, vomiting | K21.9 gastro-oesophageal reflux disease without oesophagitis  K90.4 malabsorption due to intolerance, not elsewhere classified | 1 |
| Diarrhoea, immunosuppression | B34.9 viral infection, unspecified  R21.X rash and other nonspecific skin eruption  R50.9 Fever, unspecified | 1 |
| Cyanosis/pallor | R23.0 Cyanosis | 1 |
| Apnoea | J98.8 Other specified respiratory disorders | 1 |
| Adrenal suppression | E27.4 Other and unspecified adrenocortical insufficiency | 1 |
| Abdominal pain | R10.4 Other and unspecified abdominal pain | 1 |
